# Cognitive landmark research beyond visual cues using GIScience

**DOI:** 10.3389/fpsyg.2023.1092715

**Published:** 2023-03-22

**Authors:** Kai Hamburger, Eva Nuhn

**Affiliations:** ^1^Experimental Psychology and Cognitive Science, Department of Psychology, Justus Liebig University, Giessen, Germany; ^2^Geoinformatics Group Augsburg, University of Augsburg, Augsburg, Germany

**Keywords:** landmarks, wayfinding, visual, auditory, olfactory, cognition, GIScience

In human spatial orientation, especially in wayfinding and navigation, landmarks play a pivotal role (for a review see Richter and Winter, 2014; Yesiltepe et al., 2021). This notion has been challenged by Montello (2017), claiming that the significance of landmarks in wayfinding and navigation is overestimated. Presson and Montello (1988) stated earlier that “a landmark is any distinct object or feature that is noticed and remembered” (p. 378). As a result, the focus in landmark-based wayfinding research was in the past mainly on *visual* object features (Sorrows and Hirtle, 1999; Raubal and Winter, 2002; Nuhn and Timpf, 2017a). Then, however, the observer has been put more into focus by Caduff and Timpf (2008) with their trilateral connection between landmark, environment, and the observer. But still, (visual) landmark features remained in the center of this research field. This more observer-centered approach was further established by Nuhn and Timpf (2017b), who suggested personal dimensions for landmarks. Such an approach needs to include cognitive abilities as well as subjective preferences of the observer.

We suggest that the focus in landmark-based wayfinding research needs to be shifted from an isolated “(visual) object-feature approach” to a more “individual-centered approach,” were perceptual and cognitive abilities, experiences, knowledge, and conscious vs. unconscious processing are considered as well (e.g., Ligonnière et al., 2021). This is not to say that (visual) object features are irrelevant, but it is to say that a combination of both approaches is desirable: landmark object/environmental features combined with individual capabilities, preferences, and the circumstances of the situation, in dependence of which type of information/cognitive process is available. For instance, the most trivial example to be mentioned here is the fact that we are all aware that sightless people cannot make use of visual information. This idea also accounts for people with certain types of other sensory deficit or decline, dementia, and the like (for a recent opinion article on design guidelines for elderly people which is also solely based on visual aspects, see Wiener and Pazzaglia, 2021).

Even though we are interested in how an average person finds her way through the environment, it has to be made clear that “the average wayfinder” does not exist. Thus, we may take information and processes into account, which are useful to a high degree or are very likely to fit for most people. However, in the end, we need to take a very careful look at the individual herself in order to provide valuable information for orientation, based on subjective experiences and knowledge, personal preferences, and strategies.

With regards to different modalities, recent research has additionally taken auditory as well as olfactory information into account (e.g., Karimpur and Hamburger, 2016; Hamburger and Knauff, 2019). At first glance, one might readily accept the usefulness of auditory information under certain circumstances, for instance, auditory beacons for firefighters in smoked surroundings where they can not see (Walker and Lindsay, 2006), warning signals, or signals for visually-impaired people. Additionally, Baus et al. (2007) already successfully implemented auditory signals in pedestrian navigation systems. Thus, concerning auditory signals many of us could readily accept that such information could be valuable for spatial orientation, independent of the situation.

However, many would initially doubt that olfactory information could play a significant role in normal, unimpaired spatial orientation. The general belief or the notion that humans possess a bad sense of smell goes back to Paul Broca and has survived ever since (McGann, 2017). But, McGann has shown that this is a nineteenth century myth and in the meantime it has also been demonstrated by Bushdid et al. (2014) that humans can differentiate far more odors than auditory stimuli or visual ones. It has also been demonstrated that humans are able to scent-track like dogs or rats do (Porter et al., 2007; for a very brief and more general overview, see Barrie, 2020, Chapter 11). Porter et al. could further show that humans are even capable of olfactory spatial differentiation (i.e., 3D-smelling; due to the small spatial difference between our nostrils). In conclusion, our sense of smell is far better than we often think. Therefore, it has also been claimed that one function of the olfactory system is to allow for spatial navigation (Jacobs, 2012; Jacobs et al., 2015) and that “olfaction has a major effect on how we perceive and navigate the world” (p. 611), which is actually the opening sentence of a very recent *Nature* publication by a group of archaeologists who systematically reconstruct scents from the past (Huber et al., 2022).

With regards to multisensory or multimodal processing, we are all familiar with situations in which we remember certain noises/tones and smells/odors in association with the spatial surrounding (e.g., the ice cream parlor of a vacation some years ago which was close to the beach). Thus, we should accept that spatial orientation is a multimodal process, which should not be based on isolated sensory features from one modality (as basic research normally does in laboratory experiments; see also the research bias toward vision).

The above descriptions bring about two important aspects for future research in order to reach a more comprehensive understanding of human (landmark-based) wayfinding/navigation:

1. The research focus may not just be on visual landmark features/objects but must also address other sensory modalities—even if auditory and olfactory information turn out to be of marginal or even no use in normally-sighted people;
2. (Subjective) cognitive processing mechanisms need to be taken into account, such as deliberate versus incidental learning of environmental information as well as the differentiation whether a person is familiar with an environment or not (e.g., Hamburger, 2020), and which type of information is preferred by the observer, amongst others.

All that has an impact on how we represent our surroundings and how we ultimately navigate the world.

Therefore, we here want to emphasize the following:

1. It is possible to (actively/explicitly) perceive and remember auditory and olfactory information in our spatial environment and
2. These information can be systematically mapped for further use (e.g., route descriptions).

On the first issue, we already have data on different modalities available from our own research and from other groups. For the second point we made use of a Geoinformationsystem (GIS), which provides several functionalities suitable for our project, ranging from data collection to data management and data analysis to data presentation (Bill, 2016). It supports in data collection for sounds and smells, then carrying out analysis with these data (e.g., determining regions for sounds and smells) and presenting the results in a suitable form (e.g., *via* a web map). Using such combined approaches further help us to identify which information are useful for spatial orientation and should also be implemented in navigation systems or not (i.e., it could also turn out that auditory and/or olfactory information are useless in certain situations/contexts).

A method that appeared in the last years in social science research to record things of the environment are so-called sensewalks (Quercia et al., 2015). These are qualitative methods of exploring the physical and cognitive experience of urban environments (Henshaw and M Cox, 2009). One early example of a sensewalk was reported in 1969, and was undertaken by Southworth (1969). In this context, he introduced the term “soundscape,” describing the acoustic environment. In 1985, Porteous (1985) introduced the term “smellscape,” describing the olfactory environment. In two studies, students of Augsburg University recorded the soundscape and the smellscape during a sound- and a smellwalk, respectively, in the city center of Augsburg. As basis for the recording, a conceptual data model was developed, in which the various object classes that are necessary for the distinction of several smells and sounds were defined. The data were collected by the students using ArcGIS Collector (Collector, 2022) to record locations of sounds and smells and microphones to record sounds. The recorded data was stored in a geodatabase to make them available for data analysis. Using the GIS, e.g., analyses identifying regions of sound and smells, calculating routes passing intersections with sounds and/or smell, or determining street intersections which are located in a region of sound or smell are possible. Figure 1 (left) shows the results for auditory information aggregated to polygons. We can see that particularly at popular places, such as Königsplatz and Rathausplatz, there exist several sound regions. In addition, these are places which host visually salient landmarks as well. This first evaluation also reveals that there are several sounds around, which could be used for navigation as well. Figure 1 (right) shows the availability of sounds and smells at the street intersections. There are street intersections for which we found either sound or smell, or even both types of possible landmark information were obtained. At these street intersections these olfactory and auditory cues are a valuable source for additional information, especially, e.g., for visually-impaired or blind people. However, in cases when there is neither smell nor sound available, still the visual component can be considered (if valuable visual information is present). In Nuhn and Timpf (2022) for instance, we identified for one street intersection no visual landmark at all (see Figure 1, intersection 24). However, there is information about sound and smell available at this very street intersection (Figure 1), which could possibly resemble a helpful navigation aid. Thus, at least in cases where there is no visual information available, other types of sensory information could help our spatial orientation and especially spatial representation of the environment.

**Figure 1:**
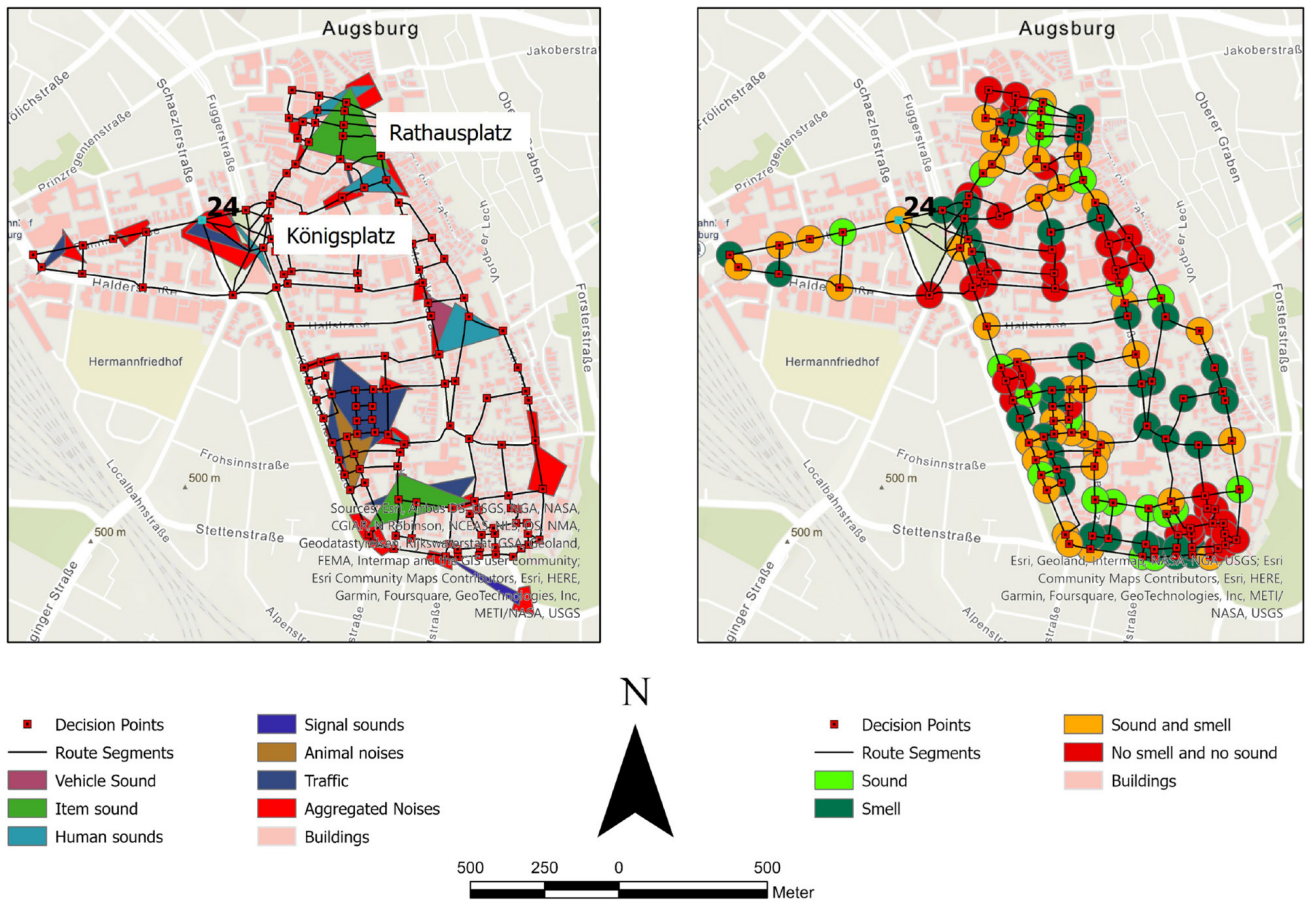
Sound regions **(Left)** and availability of sound and smells at street intersections **(Right)**.

Data and information from different or combined sensory channels can expand our mental models of the world (i.e., cognitive maps), which do not only include visual features but also sounds and smells. Therefore, such results should be used as a basis for future psychological experiments. There are several possibilities here: Empirical studies using the analyses' results for the study setup, on-site studies using the results to determine optimal routes leading participants through the real world along places with different sounds or smells (with or without additional visual information), empirical studies investigating the determined olfactory and auditory cues as landmarks in a virtual environment, as well as adaptive investigation of landmark-based wayfinding and navigation (i.e., which information is best for which person) and the mental representations thereof.

Further interesting findings from the literature are to be briefly reported at this stage:

1. Sometimes there is an overlap of information at a certain intersection/location, where multimodal representation of information could be more valuable than representation in one modality alone, e.g., Karimpur and Hamburger (2016);
2. As reported, many different sounds and smells have been found and could be differentiated (even labeled verbally, which is typically pretty difficult for smells).

These findings combine empirical results from Psychology and Geoinformation Science. Such interdisciplinary approaches need to be considered more thoroughly in the development of future personalized navigation systems. For example, for visually-impaired or blind people olfactory and auditory cues represent vital hints (Koutsoklenis and Papadopoulos, 2011a,b). Also, for people with particular interests, e.g., in street music or certain restaurants, sounds and smells could be helpful navigational aids (Nuhn and Timpf, 2017b). Thus, such a database—as the preliminary one collected by the students in combination with GIS-analyses—can help to calculate routes leading along streets and passing by intersections with the favored smells and sounds in addition to visual landmark information. The other way round, GIS-based analysis can make use of the data and identify routes to avoid malodorous areas or regions with annoying noises in future personalized navigation systems. This is not just meant to provide us with “optimal” information in human spatial orientation and the representation of space, but it is also an issue that could possibly contribute to “wellbeing” or “a sense of security” (e.g., “I'm on the right track.”) which, for instance, reduces stress.

To conclude, even though landmark-based wayfinding is mainly based on vision, in everyday life it is a multisensory and multi-representational process. This needs to be credited in basic research as well as in the development of navigation systems and artificial intelligence to support us in everyday life spatial orientation in a valuable way (i.e., combination of Psychology and GIScience).

## Author contributions

KH conceptualized the research ideas, planned the experiments, and wrote the manuscript. EN conceptualized the research ideas, planned the experiments, analyzed the data, and wrote the manuscript. All authors contributed to the article and approved the submitted version.
